# Identification and Validation of Potential Pathogenic Genes and Prognostic Markers in ESCC by Integrated Bioinformatics Analysis

**DOI:** 10.3389/fgene.2020.521004

**Published:** 2020-12-10

**Authors:** Lu Tang, Yuqiao Chen, Xiong Peng, Yuan Zhou, Hong Jiang, Guo Wang, Wei Zhuang

**Affiliations:** ^1^Department of Thoracic Surgery, Xiangya Hospital, Central South University, Changsha, China; ^2^Department of Thoracic Surgery, The Second Xiangya Hospital, Central South University, Changsha, China; ^3^Department of Neurology, Xiangya Hospital, Central South University, Changsha, China; ^4^Department of Clinical Pharmacology, Xiangya Hospital, Central South University, Changsha, China

**Keywords:** expression, long non-coding RNA, esophageal squamous cell carcinoma, next-generation sequencing, RNA-seq, bioinformatics analysis

## Abstract

Esophageal squamous cell carcinoma (ESCC) is one of the most fatal malignancies of the digestive tract, but its underlying molecular mechanisms are not known. We aim to identify the genes involved in ESCC carcinogenesis and discover potential prognostic markers using integrated bioinformatics analysis. Three pairs of ESCC tissues and paired normal tissues were sequenced by high-throughput RNA sequencing (RNA-seq). Integrated bioinformatics analysis was used to identify differentially expressed coding genes (DECGs) and differentially expressed long non-coding RNA (lncRNA) genes (DELGs). A protein–protein interaction (PPI) network of DECGs was established using the Search Tool for the Retrieval of Interacting Genes/Proteins (STRING) website and visualized with Cytoscape. Survival analysis was conducted by log-rank tests to identify “hub” genes with potential prognostic value, and real-time reverse transcription-quantitative polymerase chain reaction (RT-qPCR) was conducted to assess expression of these genes in ESCC tissues. Transwell^TM^ assays were employed to examine the migration ability of cells after knockdown of *LINC01614* expression, followed by investigation of epithelial–mesenchymal transition (EMT) by western blotting (WB). A total of 106 upregulated genes and 42 downregulated genes were screened out from the ESCC data sets. Survival analysis showed two hub protein-coding genes with higher expression in module 1 of the PPI network (*SPP1* and *BGN*) and another three upregulated lncRNAs (*LINC01614*, *LINC01415*, *NKILA*) that were associated with a poor prognosis. High expression of *SPP1*, *BGN*, *LINC01614*, and *LINC01415* in tumor samples was validated further by RT-qPCR. *In vitro* experiments show that knockdown of *LINC01614* expression could significantly inhibit the migration of ESCC cells by regulating EMT, which was confirmed by WB. These results indicate that *BGN*, *SPP1*, *LINC01614*, and *LINC01415* might be critical genes in ESCC and potential prognostic biomarkers.

## Introduction

Esophageal cancer is the sixth most fatal malignancy worldwide with an overall survival rate ranging from 15 to 25% (Lagergren et al., 2017). The two major histologic types of esophageal cancer are ESCC and esophageal adenocarcinoma (EAC), and they have distinct genetic profiles (Cancer Genome Atlas Research Network, 2017). In China, >90% of cases of esophageal cancer are ESCC (Zeng et al., 2015). A deeper understanding of the transcriptional dysregulation of ESCC is critical for predicting the prognosis, providing appropriate treatment, and improving clinical outcomes (Peng et al., 2012; Wang et al., 2012; Zhang et al., 2013; Chen et al., 2016; Huang et al., 2017).

The advent of next-generation sequencing has enabled studies on the transcriptional features of ESCC and identification of potential target genes (Tong et al., 2012; Wang et al., 2018). In Tong et al. (2012) used transcriptome data from seven ESCC samples and five non-tumor specimens to profile the transcriptional features of ESCC. Subsequently, Wang et al. (2018) depicted the “landscape” of lncRNAs and messenger (m)RNAs in ESCC using RNA sequencing (RNA-seq) data from seven pairs of tumor samples and matched normal tissues. However, RNA-seq results from different studies are often inconsistent owing to sample heterogeneity. Furthermore, the small sample sizes of those studies limits the reproducibility and reliability of their results.

In the present study, we use RNA-seq to investigate the profiles of transcriptional features of three pairs of ESCC tissues and paired normal mucosal tissues from Xiangya Hospital within Central South University (Changsha, China). Furthermore, we integrate all the public RNA-seq data from the Gene Expression Omnibus (GEO) database and The Cancer Genome Atlas (TCGA), including the GSE111011, GSE32424, and TCGA_ESCC data sets, to identify potential pathogenic genes in ESCC. A microarray data set for ESCC (GSE53625) and TCGA data set for head and neck squamous cell carcinoma (HNSCC) (TCGA_HNSCC) were used to explore the prognostic value of these “hub” genes in discovery data sets. Expression of hub genes with potential prognostic value was confirmed further by real-time reverse RT-qPCR. Further *in vitro* studies were undertaken to explore the biological function and underlying mechanism of *LINC01614*, expression of which was upregulated in ESCC and which is considered to be a potential prognostic marker.

## Materials and Methods

### Ethical Approval of the Study Protocol

Ethical approval for the collection and use of all tissues was obtained from the ethics committee of the Xiangya Hospital of Central South University. Written informed consent was obtained from each patient to use his/her material.

### Patients and RNA-seq Data

Three samples of ESCC tumor tissue and paired normal mucosa tissues for RNA-seq were collected from patients who had undergone esophagectomy without neoadjuvant chemotherapy or radiotherapy at Xiangya Hospital. Specimens were taken from the center of the tumor. Paired normal tissues were taken from surgically dissected tissues ∼5 cm away from the tumor. These three pairs of tissues were snap-frozen in liquid nitrogen after surgery and before RNA extraction. The process used for RNA-seq is described in Supplementary File 1. Another 65 ESCC tumor specimens and 20 non-cancerous specimens were obtained from Xiangya Hospital for use in RT-qPCR.

Another two RNA-seq data sets, GSE111011 and GSE32424, obtained with the Illumina HiSeq 2500 and Illumina Genome Analyzer IIx platforms, respectively, were downloaded from the National Center for Biotechnology Information Sequence Read Archive^1^ with the identifiers SRP133303 (Wang et al., 2018) and SRP008496 (Tong et al., 2012). GSE111011 contained seven normal samples and seven tumor samples. GSE32424 contained five normal samples and seven tumor samples. More information about these datasets are shown in Table 1.

**TABLE 1:** Characteristics of the data sets used in this study.

Dataset	Platform	Sample size		Tumor type	Purpose
		Normal	Tumor		
Xiangya	Illumina HiSeq 3000	3	3	ESCC	Discovery
GSE111011	Illumina HiSeq 2500	7	7	ESCC	Discovery
GSE32424	Illumina Genome Analyzer IIx	5	7	ESCC	Discovery
TCGA_ESCC	Illumina HiSeq	11	82	ESCC	Discovery
GSE53625	GPL18109 (microarray)	179	179	ESCC	Validation of DECGs Survival analysis
TCGA_HNSCC	Illumina HiSeq	44	502	HNSCC	Validation of survival analysis

### Data Processing

The Xiangya Hospital data set, GSE111011, and GSE32424 were analyzed using a particular workflow. Briefly, clean reads were obtained from raw reads by removing adaptor sequences, reads with >5% ambiguous bases, and low-quality reads, and, they were then mapped and aligned to the human genome (GRCH38) using HISAT2 (Kim et al., 2015). RNA-seq data from TCGA_ESCC were downloaded from Firehose^2^. The GSE53625 data set (which was based on GPL18109 and contained 179 ESCC and 179 paired normal control samples) was downloaded from GEO. GSE32424 contained five normal samples and seven tumor samples. More information about these datasets are shown in Table 1. The RNA-seq data sets (Xiangya Hospital, GSE111011, GSE32424, and TCGA ESCC) were defined as “discovery data sets.” The “DEseq2” R package (R Project for Statistical Computing, Vienna, Austria) was used to screen out differentially expressed genes (DEGs) between ESCC and non-cancerous controls in all data sets. Then, we screened out differentially expressed coding genes (DECGs) and differentially expressed lncRNA genes (DELGs) using the criteria of | log2(fold change)| > 1 and false discovery rate (FDR) <0.01. Subsequently, the DECGs and DELGs were used to draw volcano plots using R.

To investigate the molecular function of DECGs, we used the “clusterProfiler” R package for Kyoto Encyclopedia of Genes and Genomes (KEGG) pathway enrichment analysis and for Gene Ontology (GO) analysis with respect to three domains: cellular component, biological process, and molecular function (Yu et al., 2012).

### Construction of a Protein–Protein Interaction Network and Module Analysis

The STRING database^3^ was used to construct a PPI network of DECGs and to investigate the relationships among them (Szklarczyk et al., 2015) with a medium confidence of 0.400. Cytoscape was employed to visualize the PPI network. The MCODE (Bandettini et al., 2012) Cytoscape plugin was used to identify highly interacted nodes in the subnetworks. The following parameters were set to their default values: maximum depth = 100, degree = 2, node score = 0.2, and k-core = 2.

### Survival Analysis and Validation of Hub Genes

To explore the prognostic value of DELGs and DECGs from the PPI network, log-rank survival analysis was carried out by the Kaplan–Meier method in the GSE53625 and TCGA_HNSCC data sets. X-Tile (Camp et al., 2004) was used to determine optimal cutoff points for the log-rank test.

Reverse transcription-quantitative polymerase chain reaction was undertaken using FastStart Universal SYBR Green Master (ROX) (Roche, Basel, Switzerland). Each sample was standardized by β-actin as an internal control gene. RT-qPCR parameters were 95°C for 10 min (holding stage), 40 cycles of 95°C for 15 s, and 60°C for 1 min (PCR stage), and then 95°C for 15 s and 75°C for 1 min (melt-curve stage). Data were analyzed by the comparative cycle threshold (2^–ΔΔ*Ct*^) method.

The sense and antisense primer sequences encoding BGN, SPP1, LINC01614, INC01415, NKILA, and β-actin mRNA were as in Supplementary Table 1.

### Gene Set Enrichment Analysis

We wished to further explore the different biological pathways in patients with high and low expression of target genes. GSEA was done using R 3.6.2^4^ employing TCGA_ESCC data. The annotated gene set of “H: Hallmark gene sets” downloaded from the MSigDB was used in the analysis. *P* < 0.05 was considered to denote significant enrichment.

### Cell Lines and Culture Conditions

Five ESCC cell lines (KYSE30, KYSE150, KYSE410, Eca109, and TE-1) were obtained from the Cell Bank of the Chinese Academy of Sciences (Shanghai, China). All cell lines were cultured at 37°C with 5% CO_2_ in RPMI 1640 medium (Gibco, Carlsbad, CA, United States) with 10% fetal bovine serum (FBS; Gibco).

### Transfection of siRNAs

Two small interfering RNAs (siRNA-1, siRNA-2) against *LINC01614* and a scrambled control siRNA (siRNA-NC) were purchased from Suzhou Genepharma Co., Ltd. The sequences of the siRNAs are given in Supplementary Table 2. The knockdown efficiency was tested by RT-qPCR 48–72 h after transfection.

### Transwell Assays

Transwell assays were carried out in Transwell chambers (pore size, 8 μm; Costar, Washington, DC, United States) according to the manufacturer’s instructions. In brief, cells were harvested after treatment with siRNA for 24 h or after no treatment. Then, 200 μL of serum-free medium (containing 5 × 10^4^ cells) was placed in the upper chamber of each insert, and 800 μL of RPMI 1640 medium with 20% FBS was placed in the lower chamber. After incubation for 48 h at 37°C, the remaining tumor cells inside the upper chamber were removed with cotton swabs before fixation and staining.

### Western Blotting

Cells were lysed using total protein extraction buffer (Beyotime Biotechnology, Shanghai, China). Equal amounts of lysate samples were separated by sodium dodecyl sulfate–polyacrylamide gel electrophoresis and then immunoblotted with primary antibodies and the corresponding horseradish peroxidase-labeled secondary antibodies. The procedure is described in more detail in Supplementary File 1.

### Statistical Analysis

Bioinformatics analysis was carried out using R 3.6.2. The results of the cytology experiments were analyzed using Prism 6 (GraphPad, San Diego, CA, United States). Student’s *t* test was used to compare two independent continuous variables. The unpaired *t* test was used to detect clinical samples of ESCC. The log-rank test was employed for survival analyses. *P* < 0.05 was regarded as significant.

## Results

### Identification of DEGs

**FIGURE 1:**
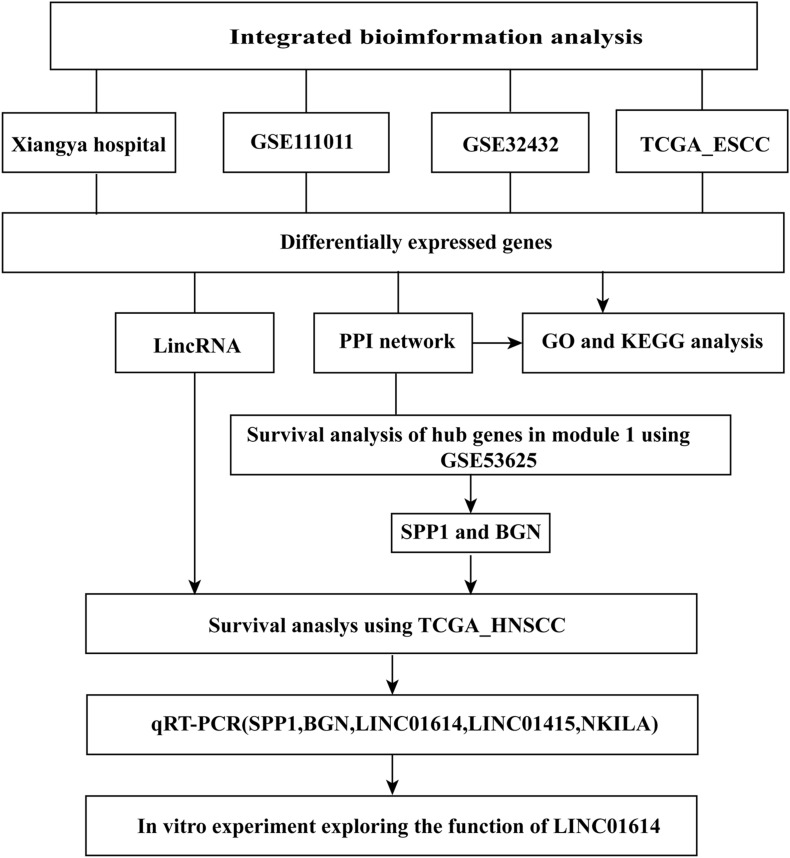
Flowchart showing the processing and analysis of data in this study.

A flowchart of the processing and analyses of data undertaken in our study is shown in Figure 1. A total of 99 ESCC tissues and 27 normal tissues from different resources (Xiangya Hospital, GSE111011, GSE32424, and TCGA_ESCC) were analyzed by RNA-seq.

**FIGURE 2:**
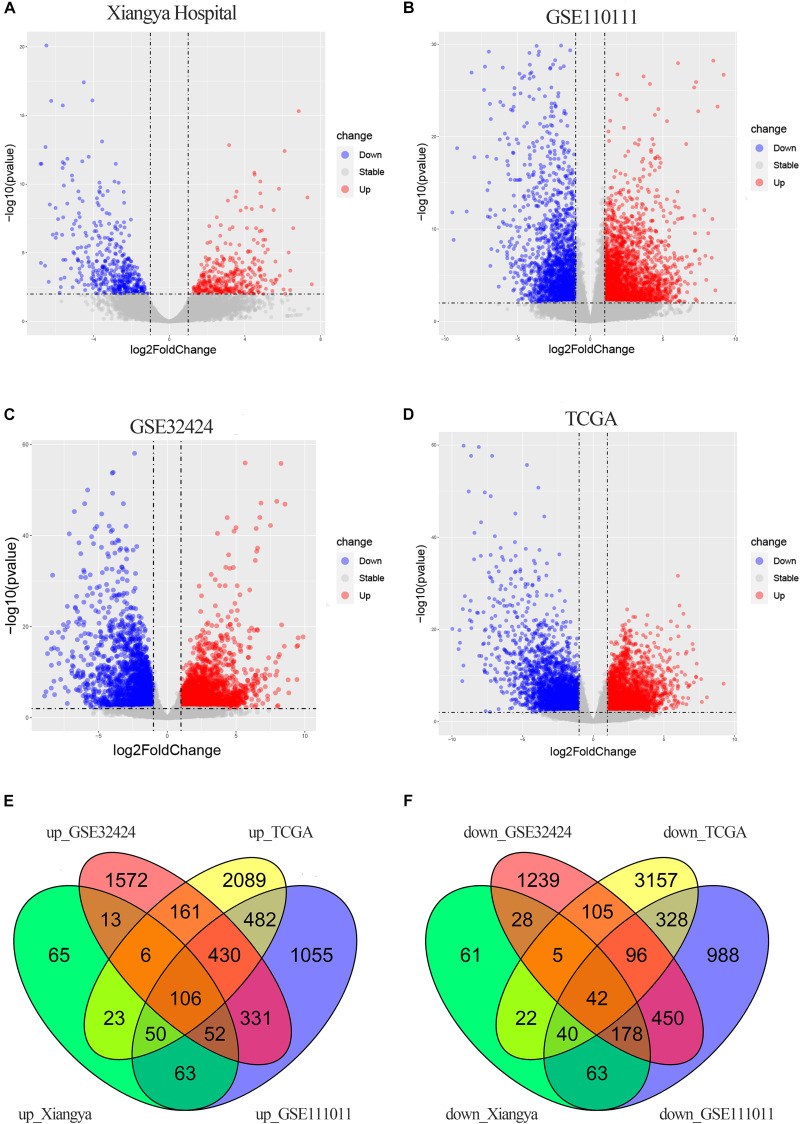
DEGs in ESCC. **(A–D)** Volcano plots showing DEGs in the discovery data sets: Xiangya Hospital **(A)**, GSE111011 **(B)**, GSE32424 **(C)**, and TCGA_ESCC **(D)**. Dark circles represent genes without significant differential expression (FDR > 0.01), red circles represent upregulated mRNAs with significant differential expression [log2(fold change) >1 and FDR < 0.01], and blue circles represent downregulated mRNAs with significant differential expression [log2(fold change) <1 and FDR < 0.01]. The top 20 upregulated and downregulated genes are listed. **(E,F)** Venn diagrams of the overlapping DEGs, including 106 upregulated **(E)** and 42 downregulated **(F)** genes, from the four data sets.

There were 816 DECGs and DELGs (377 upregulated and 439 downregulated) in the samples from Xiangya Hospital, 4754 DECGs, and DELGs (2569 upregulated and 2185 downregulated) in the GSE111011 data set, 4814 DECGs, and DELGs (2671 upregulated and 2143 downregulated) in the GSE32424 data set, and 7322 DECGs and DELGs (3347 upregulated and 3795 downregulated) in the TCGA data set. These are illustrated in the volcano plots in Figures 2A–D. As shown in the Venn diagrams in Figures 2E,F, 148 genes (106 upregulated and 42 downregulated) were consistently differentially expressed in all four databases. These commonly expressed genes are listed in Supplementary Table 3, including four upregulated genes encoding lncRNAs: *LINC01614*, *LINC01415*, *NKILA*, and *HMGA2-AS1*.

### Functional Analysis of DEGs

**FIGURE 3:**
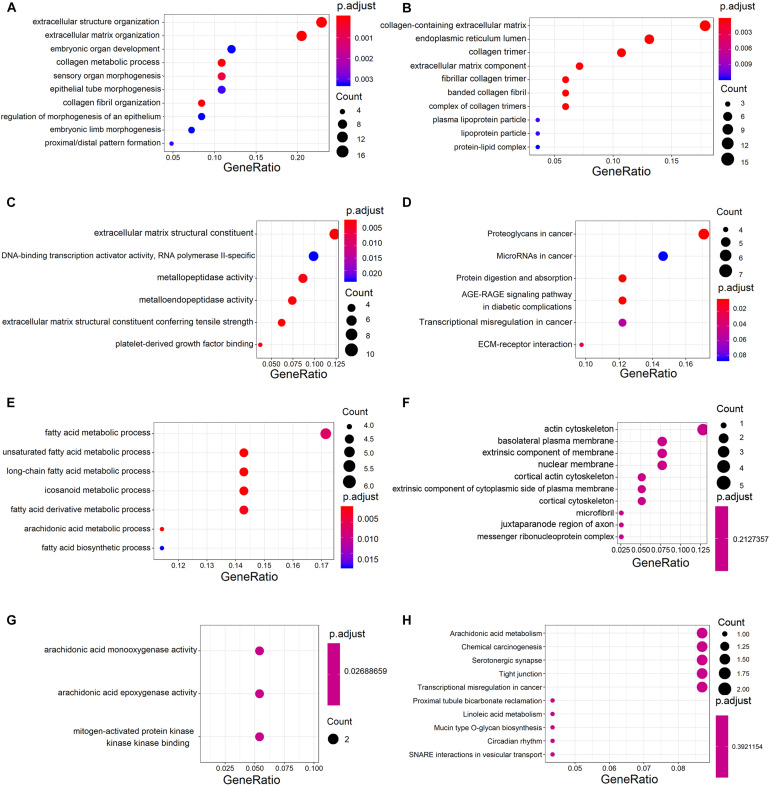
Gene ontology (GO) and Kyoto Encyclopedia of Genes and Genomes (KEGG) analysis of significant DECGs. **(A–C)** Analysis of GO annotations of upregulated DECGs with respect to three domains: biological process **(A)**, cellular component **(B)**, and molecular function **(C)**. **(D)** KEGG pathway analysis of upregulated DECGs. **(E–G)** Analysis of GO annotations of downregulated DECGs with respect to three domains: biological process **(E)**, cellular component **(F)**, and molecular function **(G)**. **(H)** KEGG pathway analysis of downregulated DECGs. The size of a dot represents the number of genes enriched for each GO term and KEGG pathway; colors from red to blue represent the adjusted *P*-value.

Analysis of GO annotations showed that the upregulated DECGs were enriched in extracellular structure organization (ontology: biological process), collagen-containing extracellular matrix (ontology: cellular component), and extracellular matrix structural constituent (ontology: molecular function) (Figures 3A–C). In analysis of the KEGG pathway, the upregulated genes were enriched significantly in proteoglycans and microRNAs in cancer (Figure 3D). Moreover, the downregulated genes were enriched in the fatty acid metabolic process (ontology: biological process), actin cytoskeleton (ontology: cellular component), and arachidonic acid monooxygenase activity (ontology: molecular function) (Figures 3E–G). According to analysis of the KEGG pathway, the downregulated genes were enriched significantly in arachidonic acid metabolism and chemical carcinogenesis (Figure 3H).

### Construction of a Protein–Protein Interaction Network, Module Analysis, and Selection of Hub Genes

**FIGURE 4:**
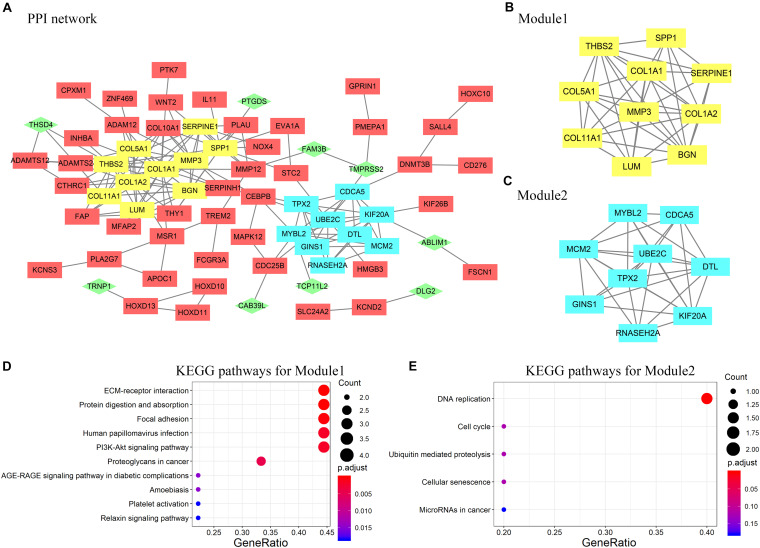
**(A)** Protein–protein interaction (PPI) network of DEGs constructed in STRING and visualized by Cytoscape. Rectangles represent genes highly expressed in ESCC tissues; diamonds and green color represent genes with low expression in ESCC tissues. **(B,C)** Two modules selected by modular analysis with the MCODE Cytoscape plugin (yellow, module 1; blue, module 2). **(D)** KEGG pathway analysis showing enrichment of module 1 genes in ECM–receptor interaction. **(E)** KEGG pathway analysis showing enrichment of module 2 genes in DNA replication. Nodes that did not interact with other nodes are hidden.

A PPI network containing 71 nodes and 172 edges was constructed by uploading 127 protein-coding genes to the STRING online database and visualized using Cytoscape (Figure 4A). In Figure 4, rectangles represent genes that were highly expressed in ESCC tissues, whereas diamonds represent genes with low expression in ESCC tissues. Subsequently, two modules were extracted using the MCODE Cytoscape plugin. Module 1 consists of 10 nodes and 29 edges (Figure 4B), and module 2 comprises nine nodes and 31 edges (Figure 4C). According to analysis of the KEGG pathway, module 1 was mainly enriched in extracellular matrix–receptor interaction and focal adhesion (Figure 4D), whereas module 2 was enriched primarily in DNA replication (Figure 4E).

### Validation of Hub Genes in Modules 1 and 2

All the hub genes in the two modules were evaluated using the GSE53625 and TCGA_HNSCC data sets. Results show that the hub genes had high expression in tumor samples in both data sets which were consistent with the results from the discovery data set (Supplementary Figure 1).

### Survival Analysis of Hub Genes in Module 1 and Differentially Expressed lncRNAs

**FIGURE 5:**
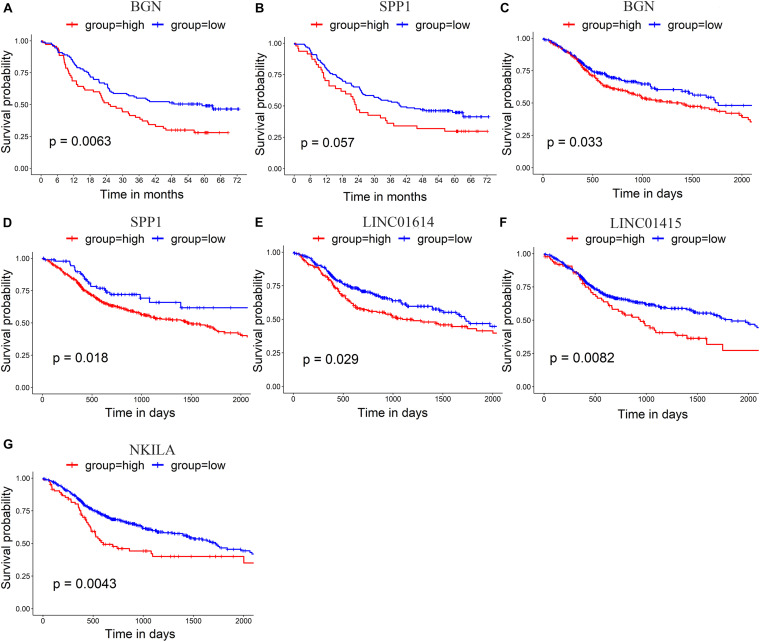
**(A,B)** Survival analysis of GSE53625 by log-rank test shows that higher expression of BGN and SPP1 is related to poor survival. **(C–G)** Survival analysis of TCGA _HNSCC by log-rank test shows that higher expression of BGN **(C)**, SPP1 **(D)**, LINC01614 **(E)**, LINC01415 **(F)**, and NKILA **(G)** is related to poor survival in HNSCC.

The prognostic value of the 10 hub genes in module 1 and the four upregulated lncRNAs was determined using the log-rank test. The best cutoff value for BGN and SPP1 in GSE53625 was 14.8 and 15.9 RPKM, respectively. The best cutoff value (in RPKM) for BGN, SPP1, LINC01614, LINC01415, and NKILA in TCGA_HNSCC was 14.1, 9.72, 3.90, 5.9, and 5.1, respectively. The results obtained using GSE53625 and TCGA_HNSCC show that higher expression of *SPP1* and *BGN* was related to worse overall survival in ESCC (Figures 5A–D) as was higher expression of three differentially expressed lncRNAs (*LINC01614*, *LINC01415*, and *NKILA*) (Figures 5E–G).

### Validation of Hub Genes in ESCC Tissue

**FIGURE 6:**
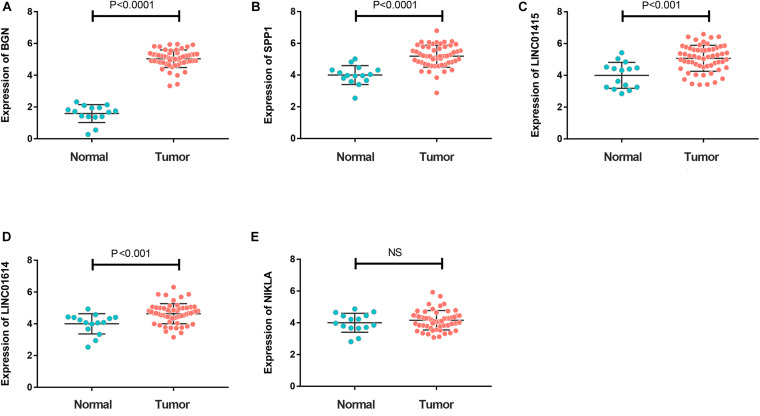
**(A–D)** Reverse transcription-quantitative polymerase chain reaction (RT-qPCR) showing much higher expression of *BGN* **(A)**, *SPP1* **(B)**, *LINC01415* **(C)**, and *LINC01614* **(D)** in ESCC tumor tissues compared with that in normal tissues (*P* < 0.05). **(E)** A similar expression of *NKILA* was observed in tumor and normal samples. The mean gene expression in normal tissue was used as a reference to calculate the value of 2^– ΔΔ*Ct*^. NC, negative control.

To further investigate expression of hub genes with prognostic value in ESCC, we undertook RT-qPCR screening of the expression of *BGN*, *SPP1*, *LINC01415*, *LINC01614*, and *NKILA* in 65 ESCC specimens and 20 non-cancerous specimens of esophageal tissue. Expression of *BGN*, *SPP1*, *LINC01415*, and *LINC01614* was much higher in tumor samples than in normal esophageal tissues (Figures 6A–D). However, there was no difference in expression of *NKILA* between tumor samples and normal samples (Figure 6E).

### Knockdown of *LINC01614* Expression Inhibits Metastasis of Esophageal Squamous Cell Carcinoma via Regulation of EMT

**FIGURE 7:**
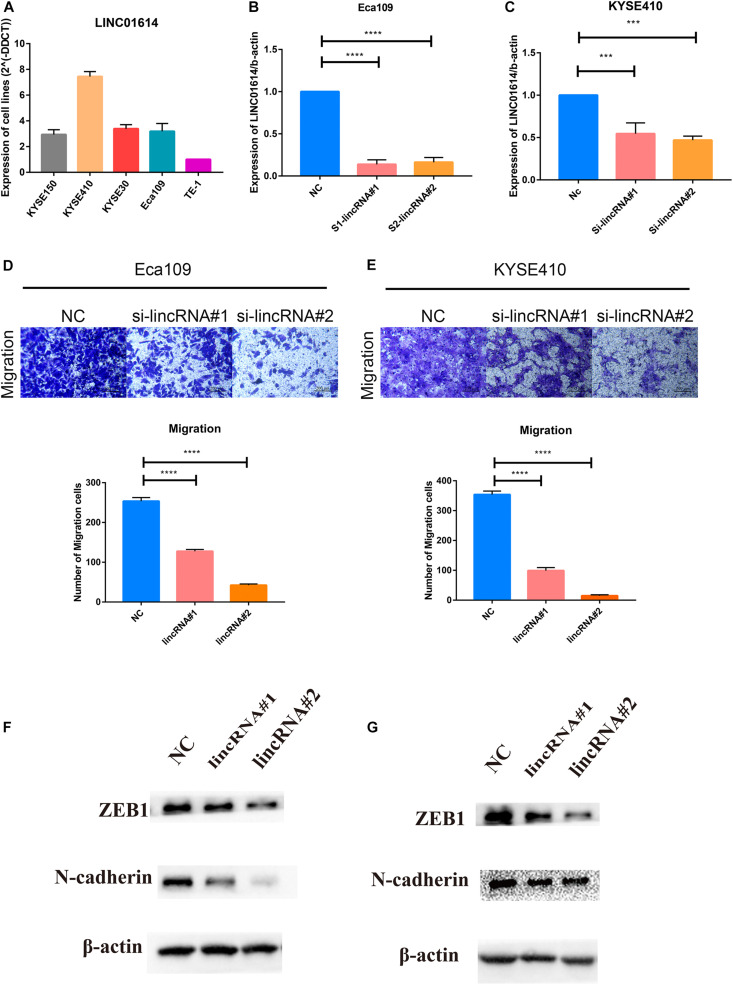
**(A)** Two cell lines (Eca109 and KYSE410) were selected for subsequent experiments due to their relatively high expression among the five candidate ESCC cell lines (KYSE150, KYSE410, KYSE30, Eca109, and TE-1). **(B,C)** si-LINC01614#1 and si-LINC01614#2 show significant knockdown efficiency. **(D,E)** Downregulation of LINC01614 expression inhibited the migration ability of ESCC cell lines (Eca109 and KYSE410). **(F,G)** Knockdown of LINC01614 expression reduced expression of N-cadherin and ZEB1 in Eca109 and KYSE410 cells. Data are the mean ± SD from three independent experiments. ***P < 0.001; ****P < 0.0001. NC, negative control.

Gene set enrichment analysis revealed that the EMT gene set was positively correlated with LINC01614 expression (NES 3.894, *P* = 0.006; Supplementary Figure 2). First, we screened *LINC01614* expression in five ESCC cell lines (KYSE150, KYSE410, KYSE30, Eca109, and TE-1) using RT-qPCR (Figure 7A). Two cell lines (Eca109 and KYSE410) were selected for subsequent experiments because they showed relatively high expression. Eca109 and KYSE410 cells were transfected with two siRNAs against *LINC01614*: si-LINC01614#1 and si-LINC01614#2, respectively. Scrambled siRNA-transfected cells were used as negative controls. si-LINC01614#1 and si-LINC01614#2 showed significant knockdown efficiency (Figures 7B,C). The results of the Transwell assay showed that the migration rate of *LINC01614* knockdown cells was less than that of control cells (*P* < 0.001, Figures 7D,E). Western blotting (WB) showed that knockdown of *LINC01614* expression reduced the expression of N-cadherin and *ZEB1* in Eca109 and KYSE410 cells (Figures 7F,G). We also explored the role of LINC01415 in ESCC: knockdown of *LINC01415* expression reduced the migration of ESCC cells without affecting EMT-related markers (Supplementary Figure 3).

## Discussion

Exploring the potential mechanisms underlying ESCC development would be of considerable benefit for prognosis prediction. In this study, 106 upregulated and 42 downregulated genes were identified in discovery data sets, including four genes encoding lncRNAs whose functions were evaluated by *in vitro* studies. The MCODE plugin of Cytoscape was used to screen out two significant modules from the PPI network of 127 protein-coding genes.

The microarray data set GSE53625 was employed to explore the prognostic value of hub genes. Of the 10 hub protein-coding genes in module 1 of the PPI, high expression of *SPP1* and *BGN* indicated a poor prognosis. BGN and SPP1 are essential components of the extracellular matrix, which has a critical role during the migration and progression of tumor cells (Rangaswami et al., 2006; Hu et al., 2016). Extensive studies have elucidated the crucial role of *BGN* in regulating the progression and metastasis of various malignancies, including prostate (Jacobsen et al., 2017), gastric (Hu et al., 2016), endometrial (Sun et al., 2016), and colon cancers (Jacobsen et al., 2017; Liu B. et al., 2018). Similarly, osteopontin (which is encoded by *SPP1*) is correlated significantly with tumor metastasis and a poor prognosis in cancers (Briones-Orta et al., 2017; Cabiati et al., 2017; Xu et al., 2017; Li et al., 2018; Zhao et al., 2019). However, the prognostic value of the four upregulated lncRNAs could not be determined owing to the deficiency of lncRNA probes in the GSE53625 microarray platform.

The TCGA_ESCC database had limited testing power because it contains data for 82 patients, only 31 of whom reached the endpoint in the follow-up. Moreover, ESCC is distinct from EAC in its genetic and epigenetic features (Cancer Genome Atlas Research Network, 2017). Therefore, we sought another approach for our ESCC research.

Moreover, a previous study suggests that the unmatched norms from healthy individuals are different from paired normal tissue, which is obtained from patients (Buzdin et al., 2018). In this study, we initially screened the DEGs between tumor tissue and paired normal tissues in the TCGA data set. Then, we also profiled the DEGs between the TCGA and GTEx databases; the latter has gene expression data obtained from healthy individuals (Supplementary Figure 4A). Results show that, when compared to normal tissue, the number of DEGs is markedly higher (Supplementary Figure 4B). However, there are still 3521 overlapped genes. Further, we detected the overlapped genes with these DEGs, which revealed that there were 110 genes that shared the common expression pattern. Notably, the five genes (LINC01415, LINC01614, NKILA, SPP1, and BGN) we selected for analysis were within the 110 genes.

According to previous studies, ESCC and HNSCC can be considered almost a single disease entity with similar molecular characteristics according to multiplatform data, including data on somatic copy number alterations, DNA methylation, and transcription (Cancer Genome Atlas Research Network, 2017). They also have the same histology type (i.e., squamous cell carcinoma) and field cancerization (i.e., upper gastrointestinal tract) and have common risk factors, including use of tobacco and alcohol (Onochi et al., 2019). Furthermore, similar expression of hub genes to that found in ESCC was detected in the TCGA_HNSCC database. Thus, we believe that combined analysis of the data for ESCC and HNSCC is a promising approach for exploring ESCC features. Furthermore, studies using TCGA_HNSCC have shown the same results as those using GSE53625 (i.e., higher expression of *SPP1* and *BGN* indicate poor overall survival in both data sets). Besides this, overexpression of *LINC01614*, *LINC01415*, and *NKILA* was related to a poor prognosis in HNSCC and ESCC.

To verify the reliability of these bioinformatics evaluations, we carried out RT-qPCR to assess expression of hub genes (*BGN*, *SPP1*, *LINC01614*, and *LINC01415*) in clinical tumor samples. These genes had much higher expression in tumor tissues than that in normal tissues data that were consistent with the bioinformatics results.

*NKILA* is a nuclear factor kappa light-chain enhancer of activated B cell (NF-κB) interacting lncRNA. It has been shown to function as a tumor suppressor by inhibiting the NF-κB pathway in various cancers, including breast cancer (Liu et al., 2015; Wu et al., 2018), melanoma (Bian et al., 2017), non-small cell lung cancer (Lu et al., 2017), nasopharyngeal carcinoma (Zhang et al., 2019), laryngeal cancer (Yang et al., 2018), and ESCC (Lu et al., 2018).

Recent studies show that *LINC01614* has high expression in tumor tissues and is associated with a poor prognosis in breast cancer (Wang et al., 2019) and non-small cell lung cancer (Sun and Ling, 2019). *LINC01614* also promotes the carcinogenesis of lung adenocarcinoma by downregulating expression of microRNA-217 and upregulating expression of Forkhead Box Protein P1 (FOXP1) (Liu A. N. et al., 2018). However, the role of *LINC01614* in ESCC is not known. We show that inhibition of *LINC01614* expression (i) by siRNA could significantly suppress migration of ESCC cells and (ii) in ESCC cell lines could decrease expression of EMT markers, including N-cadherin and ZEB1. In summary, *LINC01614* is a novel oncogene in ESCC with a critical role in the metastasis of ESCC cells.

Overall, by integrated bioinformatic analysis of transcriptome data, we identified two upregulated lncRNAs (*LINC01614* and *LINC01415*) and two hub protein-coding genes (*SPP1* and *BGN*) as potential pathogenic genes and prognostic markers in ESCC. Moreover, GSEA revealed that the EMT gene set was positively correlated with LINC01614 expression. *In vitro* experiments revealed that knockdown of *LINC01614* expression suppressed the migration ability of ESCC cell lines via EMT regulation.

However, our study is limited by an insufficient number of samples and loss of patients to follow-up. Besides this, the regulatory mechanism of *LINC01614* in ESCC is not known. Consequently, further clinical data and additional basic research are required to explore the role of *LINC01614* in ESCC.

## Data Availability Statement

Publicly available datasets were analyzed in this study. This data can be found here: https://www.ncbi.nlm.nih.gov/sra/PRJNA594797, https://www.ncbi.nlm.nih.gov/sra/SRP133303, https://www.ncbi.nlm.nih.gov/sra/SRP008496, https://gdac.broadinstitute.org, and https://www.ncbi.nlm.nih.gov/geo/query/acc.cgi?acc=GSE53625.

## Ethics Statement

The studies involving human participants were reviewed and approved by the Ethics Committee of the Xiangya Hospital of Central South University. The patients/participants provided their written informed consent to participate in this study.

## Author Contributions

GW and WZ contributed to conception and design of the study. YZ organized the database. XP performed the statistical analysis. YC and LT wrote the first draft of the manuscript. HJ wrote the sections of the manuscript. All authors contributed to manuscript revision, read and approved the submitted version.

## Conflict of Interest

The authors declare that the research was conducted in the absence of any commercial or financial relationships that could be construed as a potential conflict of interest.

## Footnotes

1 https://www.ncbi.nlm.nih.gov/sra

2 https://gdac.broadinstitute.org

3 https://string-db.org

4 http://www.r-project.org/

## Supplementary Material

Supplementary Figure 1 All the hub genes in modules 1 and 2 show high expression in tumor samples in GSE53625 **(A,B)** and TCGA_HNSCC **(C,D)**. These data are consistent with the results in the discovery data set. *****P* < 0.001.

Supplementary Figure 2 Enrichment plots of GSEA correlation analyses for LINC01614 with EMT-associated gene sets using TCGA_ESCC data.

Supplementary Figure 3 **(A)** Two cell lines (Eca109 and KYSE30) were selected for subsequent experiments owing to their relatively high expression among the five candidate ESCC cell lines (KYSE150, KYSE410, KYSE30, Eca109, and TE-1). **(B,C)** si-LINC01415#1 and si-LINC01415#2 show significant knockdown efficiency. **(D,E)** Downregulation of LINC01415 expression inhibited the migration ability of esophageal squamous cell lines (Eca109 and KYSE30). **(F,G)** Further investigation showed that knockdown of LINC01415 expression could reduce expression of N-cadherin and ZEB1 in both cell lines (Eca109 and KYSE30). Data are the mean ± SD from three independent experiments. **P* < 0.05; ***P* < 0.01; ****P* < 0.001; *****P* < 0.0001; NC, negative control.

Supplementary Figure 4 **(A)** Volcano plots of TCGA and GTEx databases. **(B,C)** Venn diagrams of the overlapping DEGs.
