# Supplementary figures and images for: Identification and Validation of Potential Pathogenic Genes and Prognostic Markers in ESCC by Integrated Bioinformatics Analysis
Source: Front Genet. 2020 Dec 10;11:521004. doi: 10.3389/fgene.2020.521004 (PMC7758294; doi:10.3389/fgene.2020.521004)

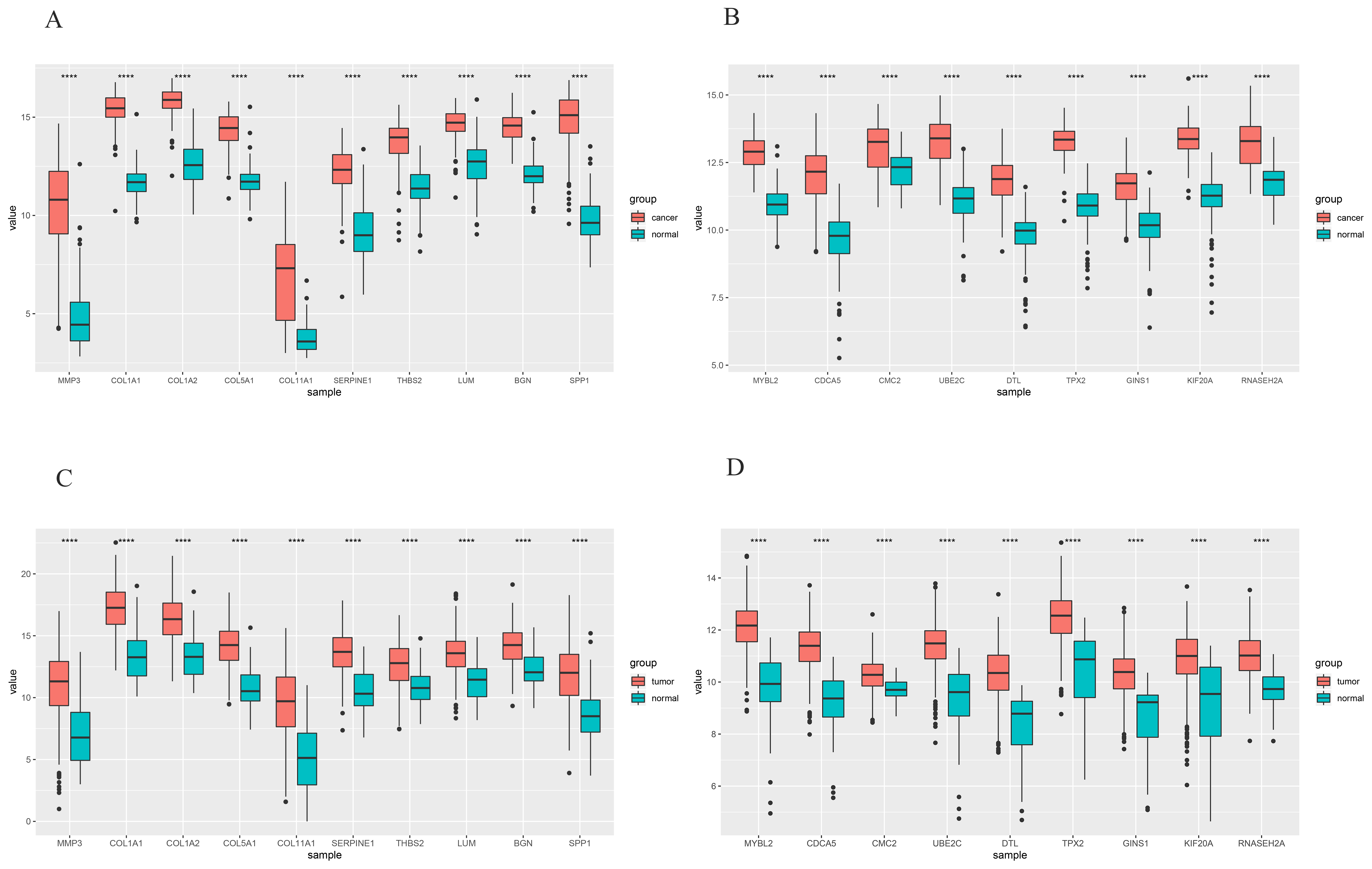

Supplement: Supplementary Figure 1 — All the hub genes in modules 1 and 2 show high expression in tumor samples in GSE53625 (A,B) and TCGA_HNSCC (C,D). These data are consistent with the results in the discovery data set. ****P < 0.001. [file Image_1.tif]

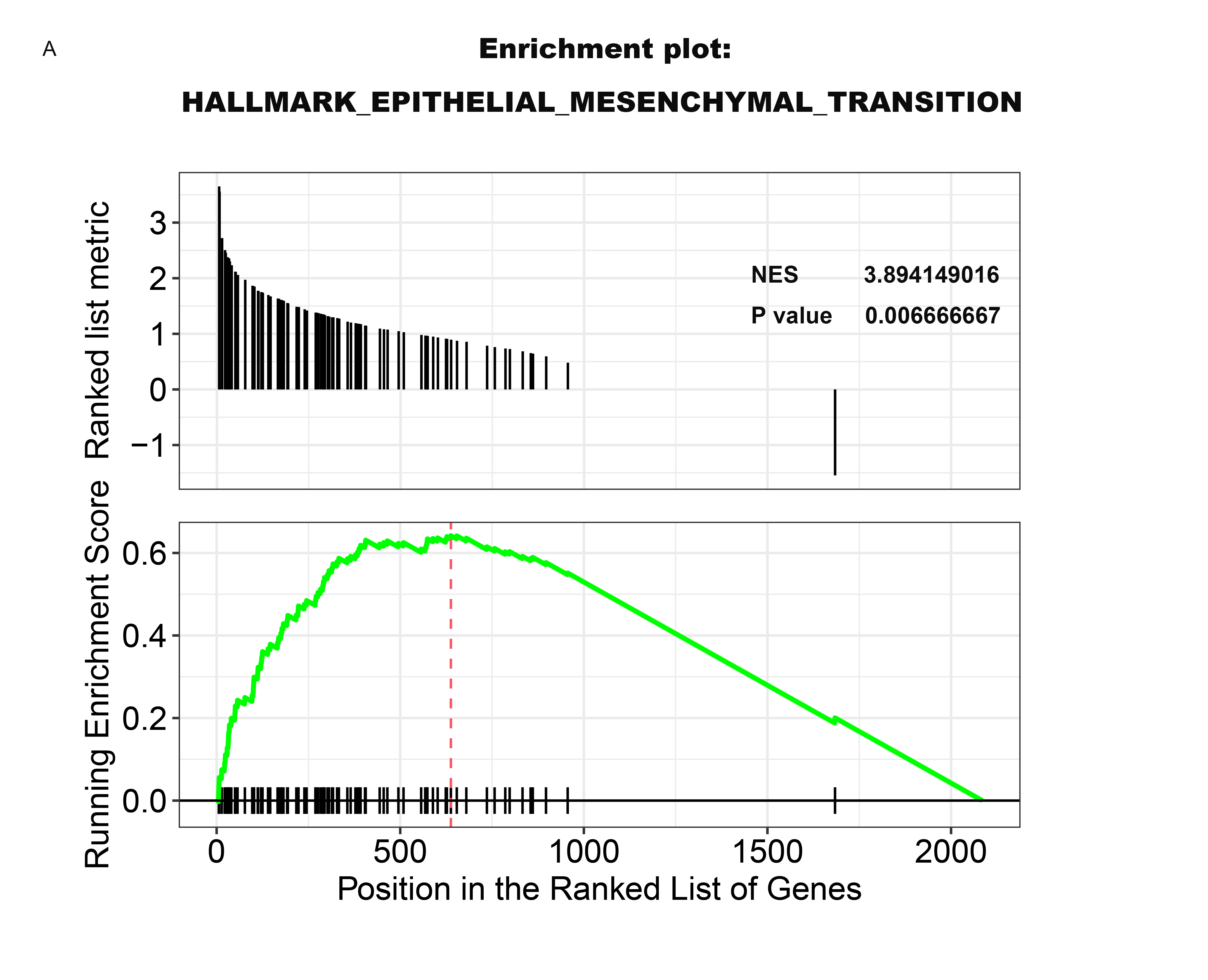

Supplement: Supplementary Figure 2 — Enrichment plots of GSEA correlation analyses for LINC01614 with EMT-associated gene sets using TCGA_ESCC data. [file Image_2.tif]

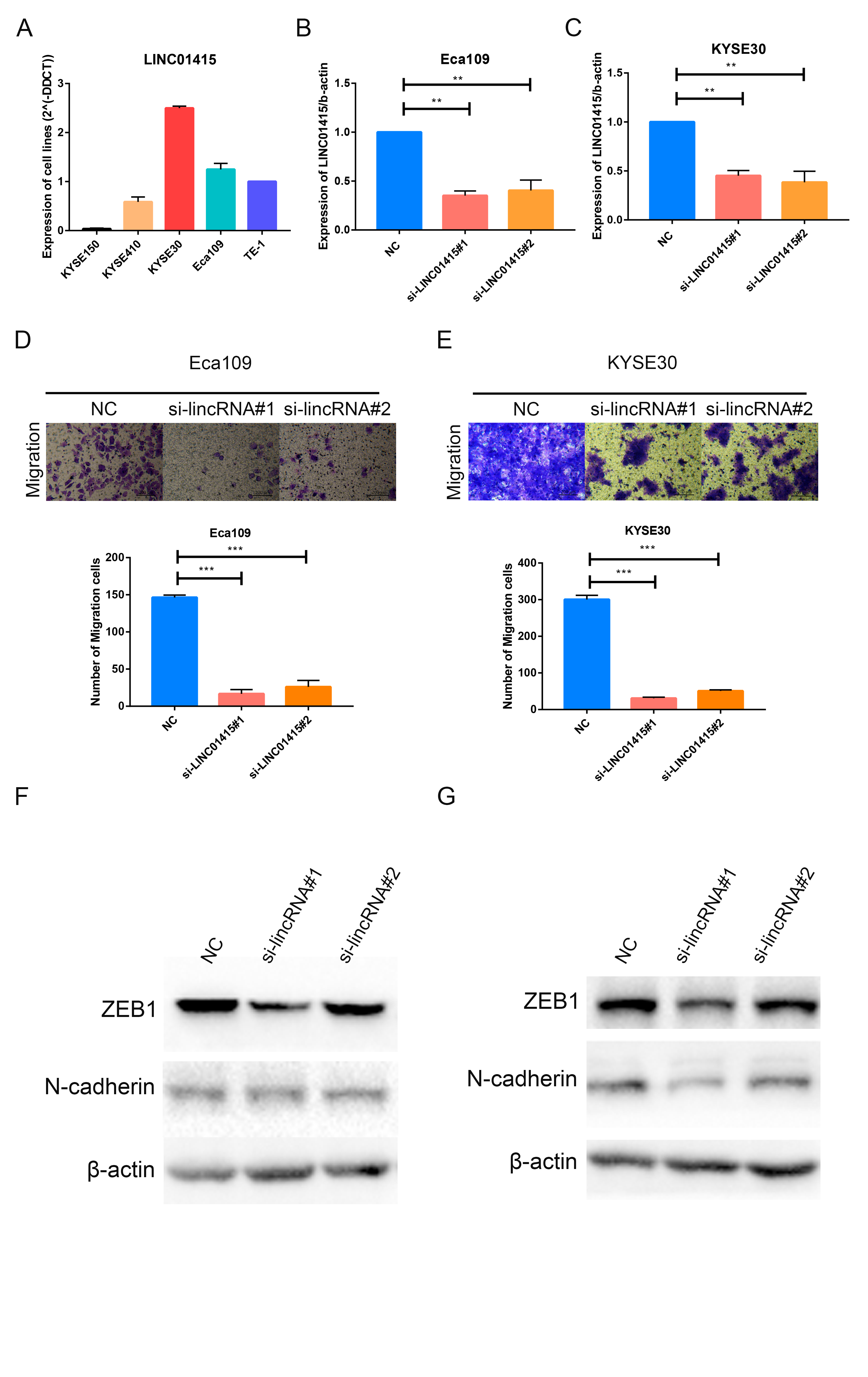

Supplement: Supplementary Figure 3 — (A) Two cell lines (Eca109 and KYSE30) were selected for subsequent experiments owing to their relatively high expression among the five candidate ESCC cell lines (KYSE150, KYSE410, KYSE30, Eca109, and TE-1). (B,C) si-LINC01415#1 and si-LINC01415#2 show significant knockdown efficiency. (D,E) Downregulation of LINC01415 expression inhibited the migration ability of esophageal squamous cell lines (Eca109 and KYSE30). (F,G) Further investigation showed that knockdown of LINC01415 expression could reduce expression of N-cadherin and ZEB1 in both cell lines (Eca109 and KYSE30). Data are the mean ± SD from three independent experiments. *P < 0.05; **P < 0.01; ***P < 0.001; ****P < 0.0001; NC, negative control. [file Image_3.tif]

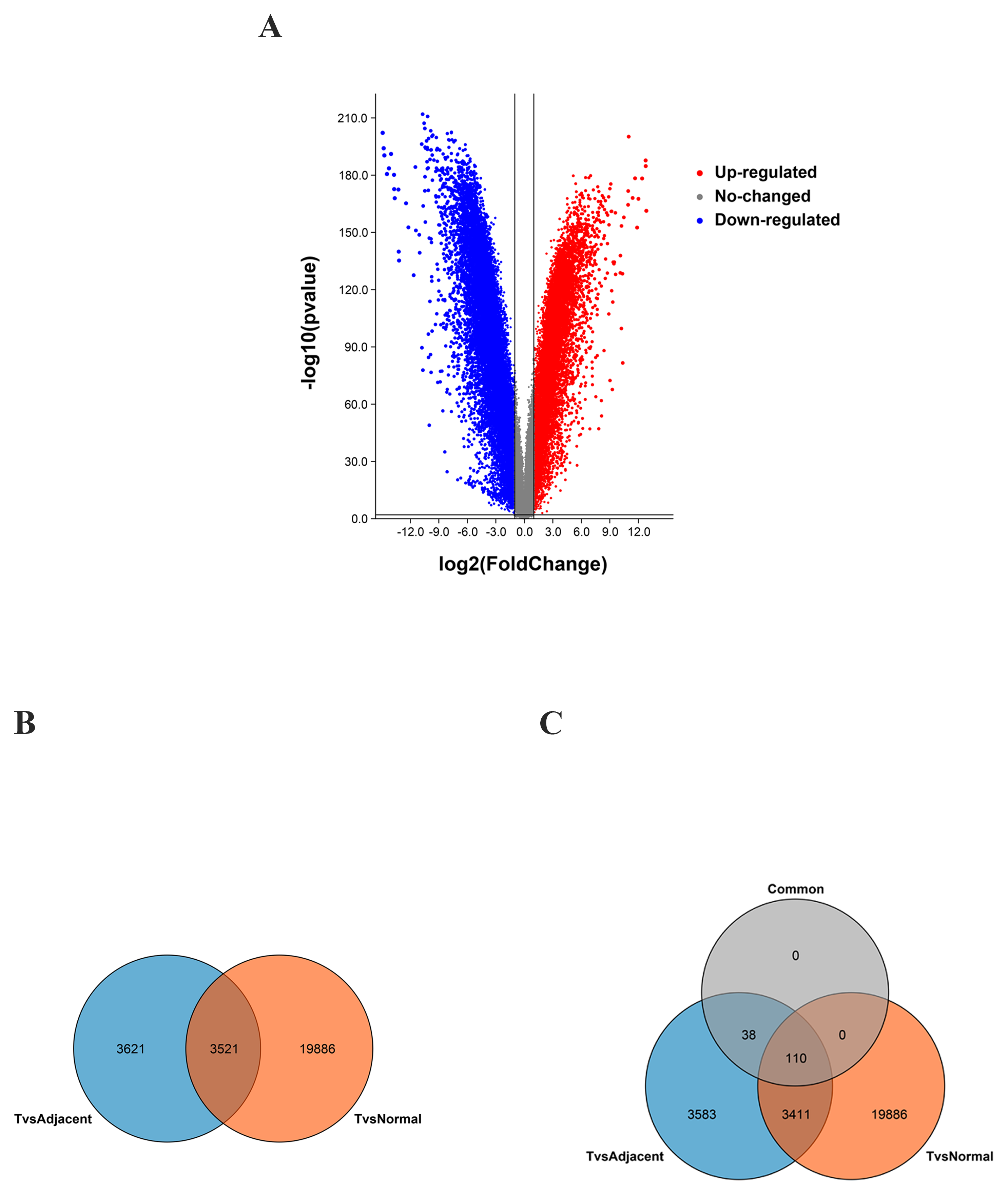

Supplement: Supplementary Figure 4 — (A) Volcano plots of TCGA and GTEx databases. (B,C) Venn diagrams of the overlapping DEGs. [file Image_4.tif]
