# Supplementary material for: Identification and Validation of Potential Pathogenic Genes and Prognostic Markers in ESCC by Integrated Bioinformatics Analysis
Source: Front Genet. 2020 Dec 10;11:521004. doi: 10.3389/fgene.2020.521004 (PMC7758294; doi:10.3389/fgene.2020.521004)
Supplement: Supplementary file 5 [file Data_Sheet_1.docx]

Preparation for whole transcriptome library and sequencing

According to the manufacturer's protocol, the total RNA was extracted using the TRIzol reagent (Invitrogen, Carlsbad, CA, USA). The RNA purity was assessed using ND-1000 Nanodrop (Thermo Scientific Inc., Santa Clara, CA, USA). The A260:A280 ratio of each RNA sample should >1.8 and the A260:A230 ratio >2.0. The integrity of each RNA sample was evaluated by the Agilent 2200 Tape Station (Agilent Technologies, Wilmington, DE USA) and make sure that the RNA Integrity Number (RIN) above 7.0. Firstly, Epicentre Ribo-Zero rRNA Removal Kit (Illumina, San Diego, CA, USA) was used to remove the rRNAs from Total RNA, and then the RNAs were fragmented into about 200bp in length. Following that, the RNAs were subjected to cDNA synthesis, according to the manufacturer's instructions, NEB Next Ultra Directional RNA Library Prep Kit for Illumina (NEB， Ispawich， USA) was used for adaptor ligation and enrichment. After diluted to 10 pM, the prepared library products were then delivered to in situ cluster generation on the pair-end flow cell. The RNA libraries were sequenced (2×150 bp) on the HiSeq3000 (Illumina, San Diego, CA, USA ).

Western blotting

Cells were lysed by a total protein extraction buffer (Beyotime Biotechnology, Shanghai, People's Republic of China). Equal amounts of lysate samples were then separated by SDS-PAGE and immunoblotted with primary antibodies and corresponding HRP-labeled secondary antibodies. Briefly, total proteins were extracted from cells treated with 100 nM siRNA for 48 and 96 h, and quantified using a BCA Protein Assay Kit following the manufacturer’s instruction (TianGEN, China). A total of 50 μg proteins was denatured, loaded into an 10% SDS-PAGE, electrophoresed and transferred to PVDF membranes. The membrane was blocked in TBST buffer containing 5% non-fat milk for 2 h at room temperature, and then washed with TBST buffer and incubated with a specific primary antibody against anti-snail(1:500, sc271977, Santa, United States), anti-N-Cadherin Rabbit (1:1000, ab92536, abcom, United State), anti-ZEB1(1:1000, ab2203879, abcom, United State),β-actin (1:5000, AC-15, Sigma, United States) and (HRP)-linked anti-rabbit IgG antibody proteintech (1:5000, #SA00001-2, China), at 4°C for 16 h. Following the corresponding secondary antibody incubation for 1 h at room temperature, the signal was visualized using the enhanced chemiluminescence detection system and quantitated under the Bio-Rad ChemiDoc MP system. β-actin was used as a loading control. Each experiment was repeated at least three times.
