# Supplementary material for: Identification and Validation of Potential Pathogenic Genes and Prognostic Markers in ESCC by Integrated Bioinformatics Analysis
Source: Front Genet. 2020 Dec 10;11:521004. doi: 10.3389/fgene.2020.521004 (PMC7758294; doi:10.3389/fgene.2020.521004)
Supplement: Supplementary file 6 [file Table_1.docx]

Table S1 The sequence of primers used in the study

| Primer name | sense primer | antisense primer |
| --- | --- | --- |
| SPP1 | 5′‐TTTGTTGTAAAGCTGCTTTTCCTC‐3′ | 5′‐GAATTGCAGTGATTTGCTTTTGC‐3′ |
| BGN | 5′‐AATGAACTCCACCTAGACCACAA‐3′, | 5′‐GATGTTGTTGGAGTGCGATAGAC‐3′ |
| NKILA | 5′‐AACCAAACCTACCCACAACG‐3′ | 5′‐ACCACTAAGTCAATCCCAGGTG‐3′ |
| LINC01614 | 5′‐TGTCAACCAAGAGCGAAGCC‐3′ | 5′‐CTTGGACACAGACCCTAGCAC‐3′ |
| LINC01415 | 5′‐AATGTGCCAGCGAAGAAG‐3′ | 5′‐GTCCAAGAACCGATACAATG‐3′ |
| β-actin | 5′‐ACAGCCTCAAGATCATCAGC‐3′ | 5′‐GGTCATGAGTCCTTCCACGAT‐3′ |
