# Supplementary material for: Identification and Validation of Potential Pathogenic Genes and Prognostic Markers in ESCC by Integrated Bioinformatics Analysis
Source: Front Genet. 2020 Dec 10;11:521004. doi: 10.3389/fgene.2020.521004 (PMC7758294; doi:10.3389/fgene.2020.521004)
Supplement: Supplementary file 7 [file Table_2.docx]

Table S2 The sequences of siRNAs used in the study

| Gene name | Product ID |  | Sequences |
| --- | --- | --- | --- |
| LINC01614 | Si-LINC01614#1 |  | GCCCACCTCAAATCCTGAA |
| LINC01614 | Si-LINC01614#2 |  | CCTCCAAGGCACCAACAAA |
| Negative control | SiN05815122147 |  | CCAUGGCGCCAAUUCCAAACAGUUU |
